# Carbonic anhydrase 9 expression is associated with poor prognosis, tumor proliferation, and radiosensitivity of thymic carcinomas

**DOI:** 10.18632/oncotarget.26657

**Published:** 2019-02-12

**Authors:** Yoichi Ohtaki, Kimihiro Shimizu, Reika Kawabata-Iwakawa, Navchaa Gombodorj, Bolag Altan, Susumu Rokudai, Arito Yamane, Kyoichi Kaira, Takehiko Yokobori, Toshiteru Nagashima, Kai Obayashi, Seshiru Nakazawa, Misaki Iijima, Takayuki Kosaka, Toshiki Yajima, Akira Mogi, Hiroyuki Kuwano, Ken Shirabe, Masahiko Nishiyama

**Affiliations:** ^1^ Division of General Thoracic Surgery, Integrative Center of General Surgery, Gunma University Hospital, Maebashi, Gunma, Japan; ^2^ Department of General Surgical Science, Gunma University Graduate School of Medicine, Maebashi, Gunma, Japan; ^3^ Education and Research Support Center, Gunma University Graduate School of Medicine, Maebashi, Gunma, Japan; ^4^ Department of Molecular Pharmacology and Oncology, Gunma University Graduate School of Medicine, Maebashi, Gunma, Japan; ^5^ Department of Oncology Clinical Development, Gunma University Graduate School of Medicine, Maebashi, Gunma, Japan; ^6^ Department of Innovative Cancer Immunotherapy, Gunma University Graduate School of Medicine, Maebashi, Gunma, Japan

**Keywords:** thymic epithelial tumor, thymoma, thymic carcinoma, CA9, hypoxia

## Abstract

**Introduction:**

Thymic epithelial tumors (TETs) comprise several histologies of thymoma and thymic carcinomas (TCs), and TC frequently metastasizes and causes death. We therefore aimed here to identify key molecules closely related to prognosis and their biological roles in high-risk TETs, particularly TCs.

**Results:**

RNA sequence analysis demonstrated that hypoxia-related genes were highly expressed in TETs. The expression of the hypoxia-related gene CA9 was noteworthy, particularly in TCs. Immunohistochemical analysis revealed that CA9 was expressed in 81.0% of TCs and 20.7% of all TET samples. CA9 expression was significantly associated with Masaoka stage, WHO classification, and recurrence-free survival after tumor resection (*P* = 0.005). The down-regulation of CA9 transcription in TC cell lines by small interfering RNAs significantly inhibited CA9 expression, which inhibited proliferation and increased sensitivity to irradiation.

**Conclusions:**

CA9 expression may serve as a significant prognostic marker of TETs and therefore represents a potential target for the development of novel drugs and radiation-sensitizing therapy designed to improve the outcomes of patients with TCs.

**Materials and Methods:**

We performed comprehensive transcriptome sequencing of 23 TETs and physiologic thymic specimens to identify genes highly and specifically expressed in high-risk TETs, particulary TCs. We performed immunohistochemical analysis of 179 consecutive surgically resected TETs to evaluate the significance of the association of protein expression with clinicopathological features and prognosis. The biological significance of the most promising prognostic marker was further studied using the TC cell lines, Ty-82 and MP57.

## INTRODUCTION

Thymic epithelial tumors (TETs) are the most frequent neoplasms arising from the thymic epithelium in the anterior mediastinum. TETs represent a relatively rare tumor that accounts for 0.2%–1.5% of malignant tumors, and only 2,104 patients underwent surgery for TET in Japan in 2014 [1]. Besides tumor staging according to Masaoka [2], tumor histology is an important prognostic factor of TETs [3, 4]. TETs comprise several histologies of thymomas and thymic carcinomas (TCs) as follows: Patients with low-risk thymomas such as types A, AB and B1 have a better prognosis; high-risk thymomas occasionally recur as types B2 and B3; and patients frequently die because of TCs [3, 4]. The prognosis of patients with TETs, even in case of tumor recurrence, is significantly better compared to other tumor entities like lung cancer [5, 6]. However, while 10-year overall survival (OS) rates of all TET patients was reported as 84.7% [3], 5-year and 10-year OS rates of TC patients were reported as 60% and 40%, respectively, and cumulative incidence of recurrence rises up to 40% at 10 years [7].

Several molecularly-targeted therapies developed using gene expression studies and analysis of mutations include a KIT inhibitor, epidermal growth factor receptor tyrosine kinase inhibitors, a histone deacetylase inhibitor, and an anti-insulin like growth factor 1 receptor antibody [8–10]. However, these therapies have limited efficacy for treating patients with advanced TETs.

Next generation sequencing (NGS) technology has clarified in detail the frequency of gene alterations and gene expressions specific to diverse neoplasms, which has led to the development of new molecularly-targeted therapies. Several specific gene alterations occur on patients with TETs [11–13], and the TCGA study on TET showed that tumor mutational burden of TCs was higher than that of thymomas [14]. However, significantly mutated genes in TETs like GTF2I, HRAS, NRAS, and TP53 are not targetable [14]. Although comprehensive transcriptome sequence analysis is a potent tool to identify potential target genes, only limited data are available for TETs. Especially, hypoxia related-proteins are shown to be highly expressed in TCs, however, the details are unknown [15, 16].

Here we used NGS to perform a comprehensive gene expression analysis of TETs to identify highly expressed genes, particularly by TCs. Furthermore, we aimed to identify their biological roles in TCs. Among highly expressed genes, the gene encoding carbonic anhydrase (*CA9*) most likely serves as a biomarker, a novel therapeutic target, or both, as indicated by a comparison of protein expression levels with patients’ clinicopathological features and prognosis. The biological effects of *CA9* were studied *in vitro* using the TC-derived cell lines, Ty-82 and MP57.

## RESULTS

### Comprehensive transcriptome sequence analysis of TETs

**Figure 1:**
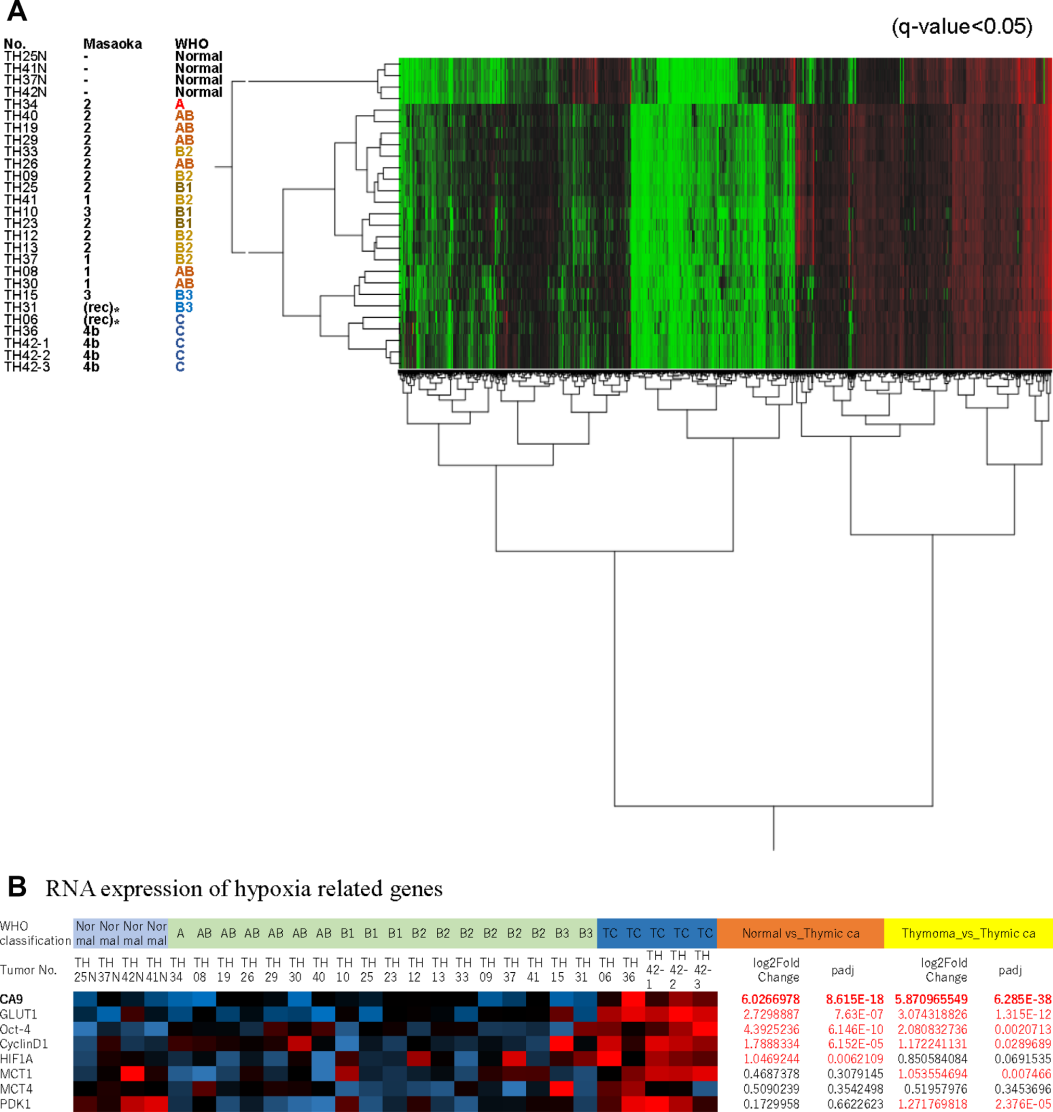
(**A**), Hierarchical Cluster Analysis of 9,200 genes differentially expressed by thymic epithelial tumors and physiologic thymic specimens. Thymic carcinoma (TC) formed a cluster distinct from thymomas. The TC subcluster was distinct but adjacent to a cluster of type B3 thymomas. (**B**), mRNA expression of hypoxia-related genes highly expressed in TCs. Among them, *CA9* was expressed at the highest levels. ^*^These samples were collected from recurrent tumors.

To determine differences in biological backgrounds, we compared the gene expression profiles acquired using NGS RNA-seq of 23 TETs and four physiologic thymic specimens (Figure 1A). Unsupervised hierarchical cluster analysis of 9,200 differentially expressed genes (DEGs) demonstrated that TCs exhibited a unique gene expression profile compared with those of physiologic thymic specimens and low-risk TETs. TCs and type B3 thymomas were classified into the same cluster, forming adjacent subclusters (Figure 1A).

We found that the expression levels of 158 genes in TCs were significantly increased compared with those in other types of thymomas and physiologic thymic specimens (log2 fold-change > 4, adjusted *P* < 0.05). As previously suggested [16, 17], metabolic or hypoxia-related genes such as *GLUT1* and *HIF1A* were highly expressed in TCs (Figure 1B), which suggested their importance in TCs, and *CA9* ranked among the top 20 highly expressed genes specific expressed in TCs (Table 1). *CA9* is a well-known gene, and it could be a good therapeutic target for thymic carcinoma. Therefore, we chose *CA9* among the top 20 candidates.

**Table 1:** Highly expressed genes in thymic carcinoma compared with thymoma and normal thymus

	Thymic carcinoma vs Thymoma		Thymic carcinoma vs Normal	
Gene names	log2 fold change	p-adj	log2 fold change	p-adj
KIT	7.372451923	6.42289E-75	6.912628497	2.487E-159
IGF2BP3	9.555242809	1.53211E-56	9.010418744	1.5625E-93
MYBPC1	11.62244454	4.11339E-55	11.61111489	1.0085E-48
LOC101929567	5.866046057	4.19853E-52	6.847485322	2.3498E-14
GFI1B	7.497300336	2.13154E-46	6.922503459	4.1378E-34
TMEM151B	7.988577363	8.83841E-46	10.8168326	2.423E-30
C12orf74	8.589187584	2.96825E-42	9.202491871	9.1126E-11
ASCL4	5.917932051	6.23545E-42	9.500790766	1.7123E-11
PLA2G3	7.705117681	2.19373E-40	5.834090367	6.4384E-13
PLEKHG7	8.611314393	1.50784E-39	11.47286352	5.2238E-19
CA9	5.870965549	6.28493E-38	6.026697828	8.6149E-18
TLDC2	5.779065153	6.28493E-38	4.315569111	7.5577E-13
MYEOV	6.750407404	2.87563E-37	9.457792251	2.4664E-39
ALOX12B	7.734309926	6.93573E-37	11.52965971	1.396E-18
KIAA1804	4.232409322	1.19054E-36	5.746459504	2.0636E-69
MFSD2A	4.780735943	9.66369E-35	4.907613797	5.9426E-11
ONECUT2	4.417990532	5.23634E-34	5.529165104	5.0619E-13
DHRS9	8.484637854	6.84171E-33	7.31756319	2.9162E-30
HOXB13	10.40352536	8.03243E-33	9.234120918	1.1286E-10
PPM1H	4.781155507	5.13178E-32	5.013480894	9.5487E-24

### Immunohistochemical analysis and prognostic significance of CA9 expression

**Figure 2:**
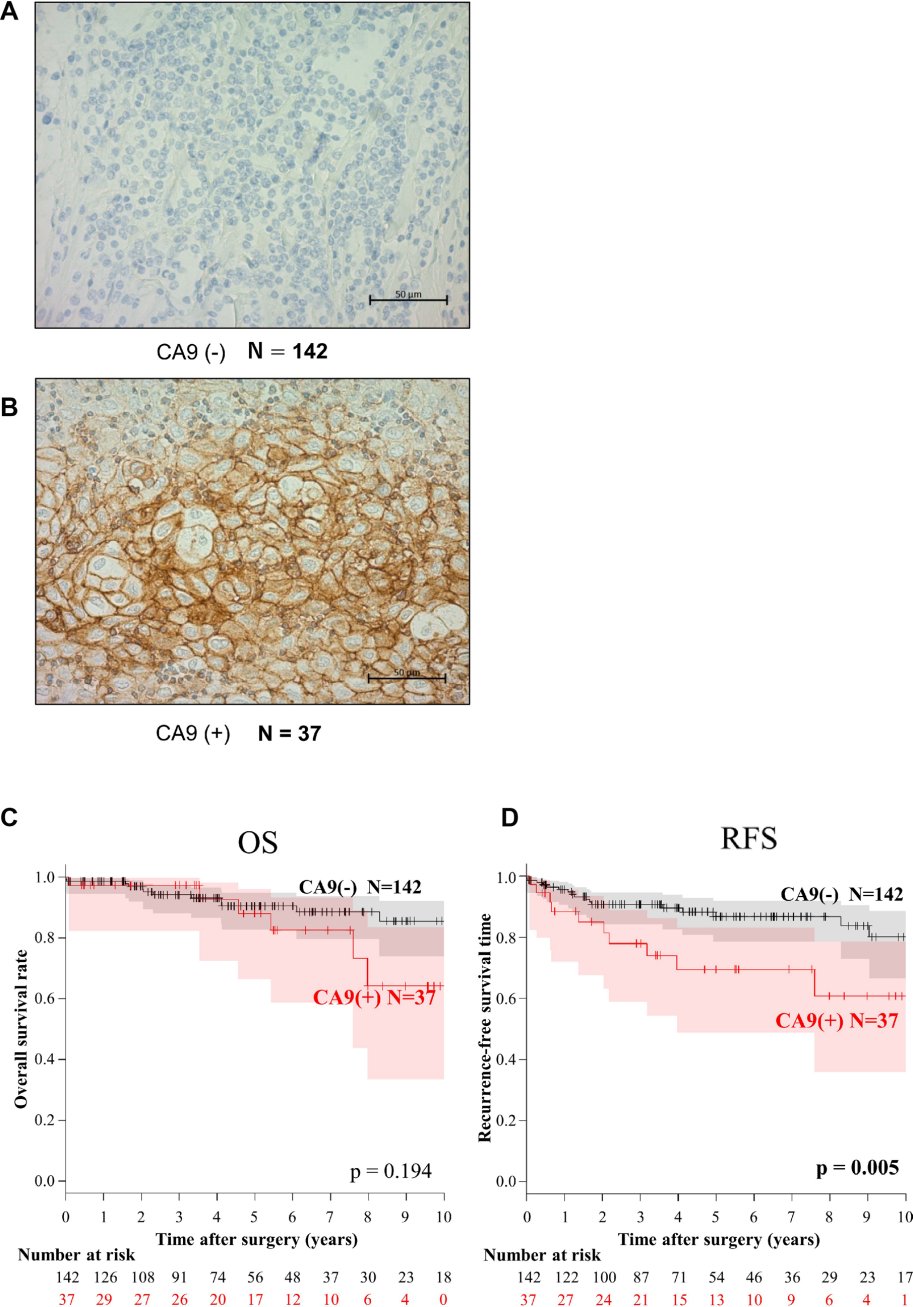
Immunohistochemical analysis of CA9 expression and the association of CA9 expression with overall survival (OS) and recurrence-free survival (RFS) of patients with thymic epithelial tumors CA9-negative (**A**) and CA9-positive thymic carcinomas (**B**). When >20% of epithelial cells were stained, the tumor was tentatively defined as CA9-positive (+). Kaplan–Meier analysis of OS (**C**) and RFS (**D**). CA9 expression significantly associated with RFS but not with OS of patients with TETs.

We next focused on the clinical significance of CA9 expression. CA9 was not detected in physiologic thymus (Supplementary Figure 1). In contrast, CA9 was detected in association with the membranes of tumor cells (Figure 2). When we tentatively classified the tumor samples into CA9-positive and -negative groups with a cutoff at staining intensity <20%, 20.7% (37/179) of all TETs were CA9-positive. Figure 2 shows representative CA9-negative (A) and CA9-positive (B) TC samples. Positive CA9 expression significantly correlated with advanced Masaoka stage (III and IV) and WHO classification (TC). CA9 expression was undetectable in type A thymomas, and only 4/93 (4.3%) of low-risk thymoma expressed CA9, whereas 17/21 (81.0%) of TCs expressed CA9 (Table 2). Although the frequency of CA9 expression by high-risk thymoma, especially type B3 tumors, was higher compared with that of *CA9* mRNA expression, the positivity of CA9 expression gradually increased according to histology, and CA9 expression data were generally consistent with its mRNA levels (Supplementary Figure 1B and 1C). We validated the mRNA values provided from NGS using RT-qPCR, and they correlated strongly with each other (Supplementary Figure 2).

**Table 2:** Correlation between CA9 protein expression and clinicopathological factors

	Total	CA9(-)	CA9(+)	
Clinicopathological factors	*N* = 179	*N* = 142	*N* = 37	*P* value
age, median (range)		58 (20–90)	53 (28–84)	0.12
gender				
male	88	67	21	0.30
female	91	75	16	
Masaoka Stage				
I	58	54	4	< 0.001^*^
II	85	69	16	
III	23	14	9	
IVa	4	2	2	
IVb	9	3	6	
WHO classification				
A	17	17	0	< 0.001^‡^
AB	37	34	3	
B1	39	38	1	
B2	40	34	6	
B3	25	15	10	
Thymic carcinoma	21	4	17	
Combined immune diseases				
Yes	52	40	12	0.61
MG	47^†^	35	12	
PRCA	4^†^	3	1	
hypogammaglobulinemia	2	2	0	
No	127	102	25	

Abbreviations: MG: Myasthenia gravis, PRCA: Pure red cell aplasia

^†^one patient had combined MG and PRCA, ^*^stage I–II vs stage III–IV, ^‡^type A-B3 vs thymic carcinoma.

Univariate analyses revealed that CA9 expression was a predictor of OS and RFS as well as the Masaoka Stage and WHO classifications (Table 3). Kaplan–Meier analysis did not indicate a statistically significant difference in OS (*P* = 0.194) between CA-positive and -negative patients with TETs, although the RFS (*P* = 0.005) of CA9-positive patients was significantly shorter compared with CA-negative patients (Figure 2C and 2D). The 5- and 10-year OS rates were 90.7% and 86.3%, respectively, of patients with CA9-negative TETs and 89.1% and 67.1%, respectively, for patients with CA9-positive TETs. In contrast, RFS of CA9-positive patients was significantly shorter compared with CA-negative patients (5-year RFS, 86.7% vs 69.4%; 10-year RFS, 80.3% vs 60.7%, respectively).

**Table 3:** Prognostic significance for overall survival and recurrence free survival (univariate analysis)

Characteristics	Number of patients (Total = 179)	5-year OS rate	*P* value^*^	5-year RFS rate	*P* value^*^
age (years)					
≤57	92	93.2	0.440	88.1	0.361
>57	87	85.5		77.5	
gender					
male	88	83.6	**0.031**^**†**^	73.4	**0.002**^**†**^
female	91	95.7		92.5	
Masaoka Stage					
I–II	143	94.6	**< 0.001**^**†**^	90.2	**< 0.001**^**†**^
III–IV	36	72.0		56.4	
WHO classification					
A–B1	93	95.1	**0.003**^**†**^	92.8	**< 0.001**^**†**^
B2–B3	65	90.5		82.4	
Thymic carcinoma	21	69.6		46.6	
Combined immune diseases					
Yes	52	89.3	0.724	89.7	0.609
No	127	89.9		79.8	
CA9 expression					
(–)	142	90.5	0.194	86.7	**0.005**^**†**^
(+)	37	88.0		69.4	

^*^Log-rank test ^†^*P* <.05.

### The role of CA9 in the proliferation and radiosensitivity of TC cells

**Figure 3:**
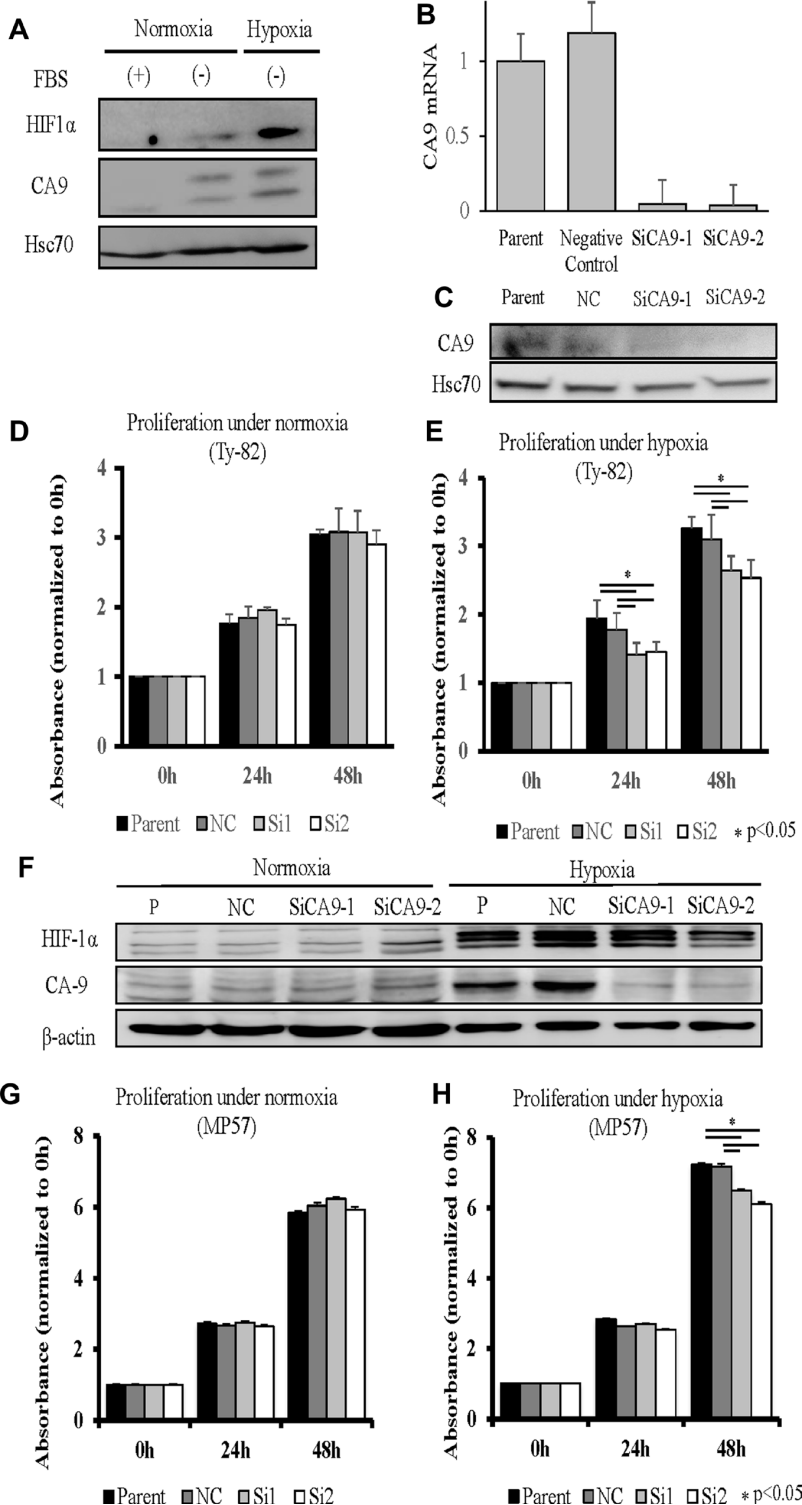
Knockdown of CA9 and its influence on the TC cell line Ty-82 (**A**–**E**). (A), Influence of hypoxia on HIF1a and CA9 expression. (B, C), RT-qPCR and Western blot analyses of the effects of CA9-specific siRNA1 and siRNA2 on CA9 expression. Knockdown of CA9 and its influence were examined on another TC cell line MP57 (**F**–**H**). (F), Western blot analyses showed both HIF1a and CA9 expressions increased under hypoxia, and CA9-specific siRNA1 and siRNA2 inhibited CA9 expression under hypoxia. The reduction in CA9 expression by the siRNAs did not inhibit cell proliferation under normoxia (D: Ty-82, G: MP57), in contrast to hypoxia (E: Ty-82, H: MP57).

CA9 expression is associated with hypoxia [18], which is consistent with our present findings that CA9 as well as HIF1a were induced in Ty-82 cells exposed to hypoxia, but not at normoxia (Figure 3A). Interestingly, cell proliferation, specifically under hypoxia, was significantly suppressed by the knockdown of CA9 expression by Ty-82 cells. *CA9*-specific siRNA1 and siRNA2 significantly inhibited the expression of *CA9* mRNA and protein (Figure 3B and 3C) as well as the proliferation of Ty-82 cells (Figure 3D and 3E) under hypoxia but not normoxia. We validated the effect using another TC cell line, MP57. Both CA9 and HIF1a were induced in MP57 cells exposed to hypoxia, but not in normoxia and *CA9*-specific siRNAs significantly inhibited the CA9 protein expression only in hypoxia (Figure 3F). The proliferation of MP57 cells was inhibited under hypoxia but not normoxia (Figure 3G and 3H).

**Figure 4:**
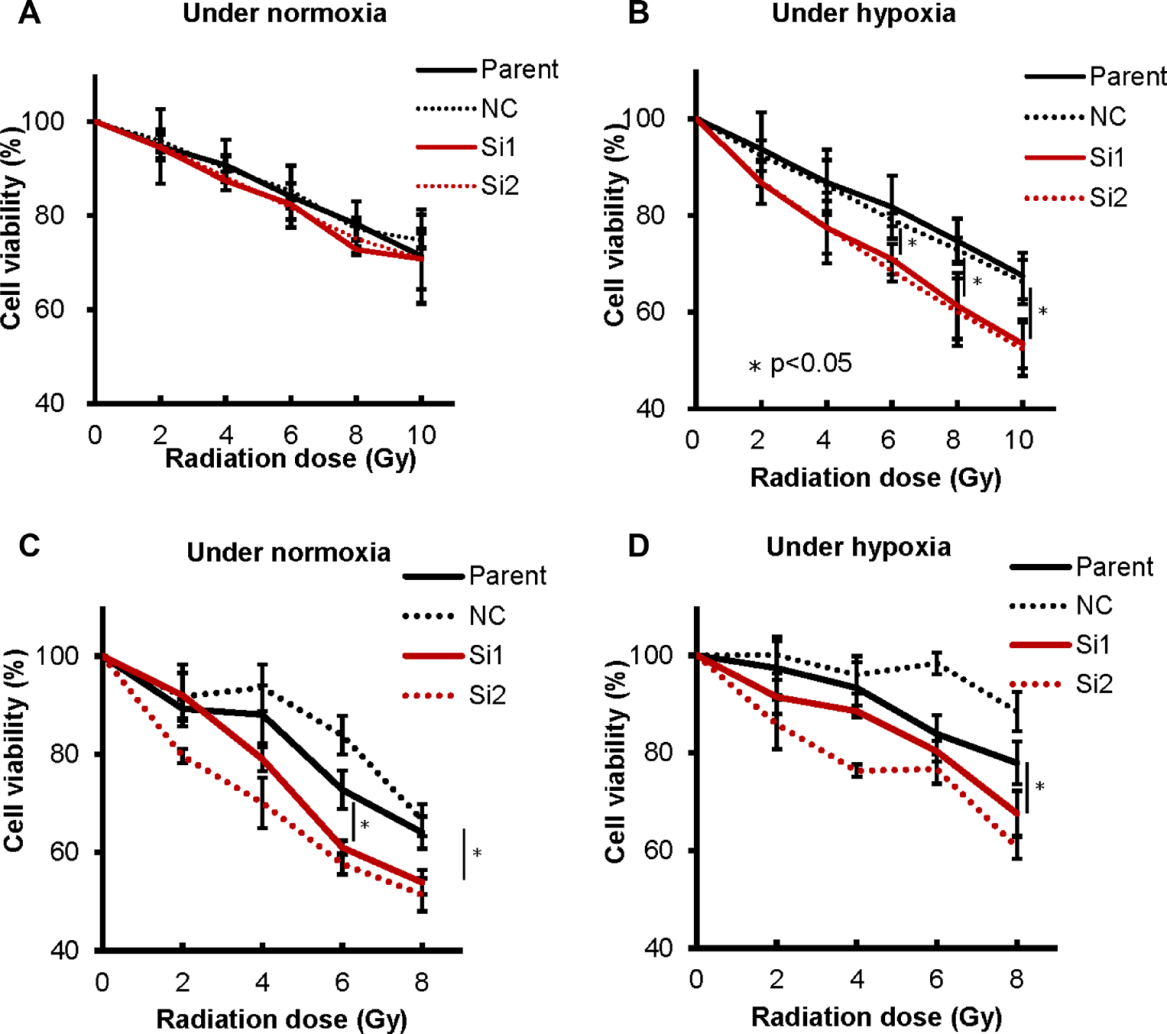
CA9 knockdown using siRNA sensitized radiation to TC cells CA9 knockdown did not influence cellular sensitivity to irradiation under normoxia (**A**), in contrast to hypoxia (**B**) in Ty-82 cells, while CA9 knockdown influenced radio-sensitivity both under normoxia (**C**) and hypoxia (**D**) in MP57 cells.

The induction of hypoxia-related factor causes cellular resistance to radiation [19]. We therefore studied the effect of CA9 on the radiosensitivity of TC cells. We found that reduced CA9 expression was significantly associated with reduced proliferation in response to irradiation under hypoxia in both Ty-82 and MP57 (Figure 4B and 4D). Although the similar combination effects of CA9 inhibition and radiation was observed in MP57 cells in normoxia (Figure 4C), the radiosensitivity of Ty-82 cells transfected with the siRNAs was not significantly altered under normoxia (Figure 4A).

## DISCUSSION

Here we conducted comprehensive gene profiling revealing that DEGs expressed by high-risk TCs and type B3 thymomas form a cluster distinct from those of low-risk TETs and B2 thymoma. TC could be classified into an adjacent subcluster that was distinct from B3 thymomas. Certain hypoxia-related genes were specifically expressed at high levels by TCs. Among them, CA9 expression of TC showed more than a 50-fold increase compared with all other TETs and physiologic thymus. Analysis of a cohort of 179 TETs shows that CA9 was expressed at significantly elevated levels in TCs, which was closely associated with clinical stage and shorter RFS of patients with TETs.

An *in vitro* study using the TC cell lines, Ty-82 and MP57 show that reduction of CA9 expression inhibited TC cell proliferation specifically under hypoxia, and increased irradiation sensitivity. These findings indicate that CA9 may play a key role in TC and may therefore serve as sensitive and specific marker of high-risk TET, as a novel therapeutic target for TC, or both.

Hypoxia is an important cause of increased tumor progression, metastasis, and resistance to anticancer drugs and irradiation [18–20]. CA9 is an isoform of the human αCA family of zinc metalloenzymes, which transport CO_2_ to the extracellular environment to regulate intracellular pH to protect cells from hypoxia and acidosis [18]. CA9 is transcriptionally regulated by hypoxia through the binding of HIF-1 to a hypoxia response element upstream from the transcription initiation site [18].

Of the CA isoforms, only CA9 and CA12 are highly expressed in cancer tissues and therefore may contribute to the acidification of the extracellular milieu [21]. CA9 expression serves as a prognostic factor of patients with the cancers as follows: lung cancer [22], breast cancer [23–25], nasopharyngeal cancer [26], esophageal cancer [27], brain tumors [28], cervical carcinoma [29], and gastric cancer [30]. Nevertheless, the roles of CA9 in TETs are unknown.

The frequency of detection of CA9 varies from 48% to 80% according to tumor site [25, 27, 29]. For example, an analysis of tissue microarrays found that CA9 serves as a diagnostic marker that distinguishes TCs from type B3 thymomas, though the diagnostic specificity and sensitivity of CA9 was not higher compared with those of glucose transporter 1 (GLUT-1) or KIT [15].

Our present study shows that CA9 was expressed in <30% of TETs, although it was expressed in >80% of in TCs. Moreover, CA9 expression was significantly associated with shorter RFS. In an effort to identify the function of CA9 in tumor cells, we found that the hypoxia-related molecules CA9 and GLUT-1 were highly expressed in TC samples. Numerous studies focus on the prognostic significance and clinical utility of 2-[^18^F]-fluoro-2-deoxy-D-glucose (^18^F-FDG) PET imaging of TETs [31–34], indicating the potential influence of hypoxia and glucose metabolism on TETs. Together, our present data revealing the biological and clinical effects of CA9 expression are therefore consistent with the findings of our comprehensive transcriptome sequence analysis that identified *CA9* as a highly significant DEG. Moreover, CA9 may play an important role in the aggressive phenotype of TET and may play a similar role in other malignancies [18, 30].

The putative roles of CA9 are as follows: regulation of intracellular pH to control the tumor microenvironment [35]; regulation of tumor growth and survival [36]; destabilization of intercellular adhesion contacts [35]; promotion of tumor migration, invasion and metastasis [37–40]; and resistance to chemotherapy and radiotherapy [41]. Extracellular hypoxia and acidosis may negatively affect drug uptake and radiation damage through the regulation of pH by CA9 [18, 42]. Here we show that inhibition of CA9 expression reduced the proliferation of TC cells, suggesting that inhibition of CA9 exerts an antitumor effect on TC. Moreover, inhibition of CA9 expression enhanced the radiosensitivity of a TC cell line, suggesting that the combination of radiation and a CA9 inhibitor may synergize to more potently inhibit the growth of TC cells. We observed that inhibition of CA9 expression using siRNA sensitized MP57 to radiation in normoxic condition, but not Ty-82. Although we have to confirm the effect using other TC cell lines in detail, inhibition of CA9 expression might have other synergic effect to improve radiosensitivity of TC cells even in normoxic condition. This possibility is consistent with the survival benefits of postoperative radiotherapy (PORT), which is often used to treat patients with TCs and thymoma [43–45]. A CA9 inhibitor might sensitize TC to PORT or other radiotherapies used to treat recurrent lesions, which will likely lead to improved prognosis of patients with TETs.

Although we extracted tumor samples from the central part of non-necrotic tissues and verified the whole tumor histology, we did not include the morphological control for molecular study. We were not able to collect early-stage TC samples, which could lead to sample bias.

In conclusion, our findings indicate that CA9 is a key molecule that shows significantly higher expression in TCs, and contributes to the aggressiveness of TCs. Moreover, CA9 expression may be involved in the proliferation and radiosensitivity of TC cells. We suggest therefore, that CA9 may serve as a therapeutic target for managing patients with TCs.

## MATERIALS AND METHODS

### Tissue samples and clinicopathological data of patients with TETs

Surgical specimens were obtained from 188 patients with TETs at Gunma University Hospital between 1991 and 2016. The study was conducted in compliance with the Declaration of Helsinki and approved by the Institutional Review Board (IRB Numbers 160116 and 2016-062). All patients provided written informed consent before their registration in the study. Twenty-three surgically resected TETs and four physiologic thymic specimens were used for RNA sequence analysis to screen for highly expressed genes specific for highly malignant TETs. Formalin-fixed, paraffin-embedded sections of tumor tissues collected from 188 patients with TETs were subjected to immunohistochemical analysis (see below). Of them, 179 samples which were not recurrent tumor were used for clinicopathological and survival analyses.

Pathological stages of the patients with TETs were determined according to the Masaoka classification [2] and WHO classifications. Patients’ clinical data were reviewed, and overall survival (OS, days) was defined as the time between the date of tumor resection and date of death from any cause or last follow-up. Recurrence-free survival (RFS) was defined as the time between the date of tumor resection and the date of any recurrence, death from causes other than cancer, or the last follow-up.

### Cell line

The human thymic carcinoma cell line Ty-82 was obtained from the Japanese Collection of Research Bioresources (JCRB) [46]. Ty-82 cells were authenticated and certified as uninfected by the JCRB at the time of purchase. We used the cells within 4 months after purchase. Ty-82 cells were cultured in a flask designed only for culturing nonadherent cell lines (Corning, NY, USA). MP57 is a human thymic carcinoma cell line kindly provided by Dr. Giuseppe Giaccone [47]. Both cells were cultured in RPMI-1640 supplemented with penicillin/streptomycin and 10% fetal bovine serum (FBS) in an atmosphere containing 5% CO_2_ in a humidified incubator at 37° C. To induce hypoxia, cells were cultured in the presence of 1% O_2_ using a BIONIX-1 hypoxic culture kit (Sugiyama-Gen, Tokyo, Japan).

### Whole transcriptome expression analysis (RNA-seq)

Total RNAs were extracted from non-necrotic central regions of 23 surgically resected TETs and four physiologic thymic specimens using a NucleoSpin RNA kit (TaKaRa Bio, Shiga, Japan). Physiologic thymic specimens were collected simultaneously from the surgically resected thymic tissue corresponding to TETs (TH25N, TH37N, TH41N, and TH42N were collected with TH25, TH37, TH41, and TH42, respectively). Fresh human specimens were collected during surgery and stored in RNAlater (Thermo Fisher Scientific) at 4 °C overnight. After removal of the RNA later reagent, samples were stored at –80° C. RNA quality was assessed using an Agilent Bioanalyzer (Agilent Technologies, Santa Clara, CA, USA), and high-quality RNAs (RNA integrity numbers >7.0) were used for RNA-seq.

Total RNA (1 µg) was used to generate sequencing libraries of barcoded fragments using the TruSeq Stranded mRNA Sample Prep Kit (lllumina, San Diego, CA, USA) following the manufacturer’s instructions. Libraries were subjected to paired-end sequencing of 43-bp reads using a NextSeq500 System (Illumina) with a NextSeq500 High Output Kit (Illumina). The reads were aligned to the UCSC reference human genome 19 (hg19) using a Spliced Transcripts Alignment to a Reference (STAR) software v2.3.1 (DNASTAR, Inc. Madison, WI, USA). DEGs were detected using DESeq v1.24.0 (Bioconductor open-development software project). We identified genes expressed at significantly higher levels in TCs compared with thymoma and physiologic thymic specimens (log2 fold-change >4, adjusted *P* < 0.05).

### Immunohistochemical analysis

Immunohistochemistry was performed using formalin-fixed, paraffin-embedded sections of TET tissues as previously described [48]. After reviewing hematoxylin and eosin-stained surgical specimens, the block containing the most extensive tumor component was selected. Sequential 4-μm sections were deparaffinized and immersed in 0.3% hydrogen peroxide in methanol for 30 min to inhibit endogenous peroxidase activity. For antigen retrieval, the slides were heated at 95° C for 45 min in distilled water with immunosaver. The slides were then incubated overnight with primary rabbit anti-human CA9 antibody (ab15086, diluted 1:1000) (Abcam, Tokyo, Japan) at 4° C in a humidified chamber. Negative-control slides were incubated without the primary antibodies, and no detectable staining was observed. Finally, the sections were counterstained using Meyer’s hematoxylin, dehydrated, and mounted. We evaluated a peritumoral tissue far from tumor as physiologic thymic specimens.

Epithelial cells with membrane staining of CA9 of any intensity above background were defined as positive. Approximately 1,000 cells were counted on each slide. For type B1 and B2 thymoma which contain a lot of lymphocytes, we counted tumor cells as much as we could. Positive detection rates were assessed according to the percentage of CA9-positive cells per epithelial cells in the samples. The tumors with ≥20% CA9-positive cancer cells were defined as CA9-positive and those with <20% were defined as CA9-negative.

### Protein extraction and Western blotting

Total proteins were extracted from Ty-82 cells using PRO-PREP (iNtRON Biotechnology, Kyungki-Do, Korea) and from MP57 cells using lysis buffer [10% Glycerol, 10mM Tris-HCl (pH7.5), 1mM EDTA, 400 mM NaCl, 0.5% NP40, 4 mg/mL aprotonin, PMSF, proteasome inhibitor MG-132 and 1mM DTT], separated using SDS-PAGE (12.5% gels), electrophoretically transferred to membranes that were then incubated overnight at 4° C with the antibodies against CA9 (ab15086, 1:2000) (Abcam, Tokyo, Japan), HIF1a (CST #3716, 1:1000) (Cell Signaling Technology, MA, USA), β-actin (CST #3700, 1:1000) (Cell Signaling Technology, MA, USA), and Hsc70 (1:4000) (Sigma, St. Louis, MO, USA). After incubation with horseradish peroxidase-conjugated secondary antibodies, immune complexes were detected using the ECL Prime Western blotting Detection System (GE Healthcare, Tokyo, Japan), and the signals were quantified using an Image Quant LAS 4000 (GE Healthcare).

### Knockdown analysis of CA9 expression and real-time RT-PCR

CA9-specific small interfering RNAs (siRNA) (ON-TARGET plus Human CA9 siRNAs J-005244-05 [siRNA1] and J-005244-06 [siRNA2]) were purchased from Dharmacon (GE Healthcare, Japan). Ty-82 and MP57 cells in OPTI-MEM were transfected with the siRNA using an electroporator (CUY21EDIT II, BEX, Tokyo, Japan) according to manufacturer’s protocol. Both cells were seeded in 2 mL of RPMI-1640 in microtiter plates and incubated with siRNAs at 37 °C for 24 h before each assay. Transfected cells were incubated for 48 h or 72h. Total RNA was extracted from cells using the RNeasy Plus Mini Kit and QIAshredder (Qiagen, Hilden, Germany), and the quantity of isolated RNA was measured using an ND-1000 spectrophotometer (NanoDrop Technologies, Wilmington, DE, USA).

The RNA was reverse-transcribed using a ReverTra Ace qPCR RT kit according to the manufacturer’s instructions (Toyobo Life Science, Osaka, Japan) in 5 µl of reaction mixture. To quantitatively compare *CA9* mRNA levels, qPCR reactions were performed using the KAPA Probe qPCR master mix (Sigma-Aldrich, St. Louis, MO, USA) with a StepOnePlus real-time PCR system (Thermo Fishier Scientific, MA, USA). The sequences of the primer pairs were as follows: CA9 forward, 5′- CCTTTGCCAGAGTTGACGAG -3′; CA9 reverse, 5′-GCAACTGCTCATAGGCACTGT -3′. Probe #25 was used. *GAPDH* mRNA was used to normalize RNA inputs (GAPDH, ABI Probe set; VIC).

### *In vitro* CCK8 assay

Ty-82 cells (4.0 × 10^3^ cells/well), MP57 cells (2.0 × 10^3^ cells/well) were plated in 96-well plates in 10% FBS–RPMI-1640) 24 h (Ty-82) or 72 h (MP57) after siRNA transfection. To induce hypoxia, cells were incubated in 1% O_2_ for 6 h (Ty-82) or 24 h (MP57) and subsequently cultured under normoxia. After 0 h, 24 h, and 48 h from transfection, 10 μL of CCK-8 solution (CCK-8; Dojindo Laboratories) was added to each well for 2 h at 37° C, and absorbance at 450 nm was determined using an xMark Microplate Absorbance Spectrophotometer (Bio Rad, Hercules, CA, USA). Proliferation rates were calculated using the absorbance at each time compared with those of the plates at 0 h.

### Radiosensitivity assay

Ty-82 cells (4.0 × 10^3^ cells/well), MP57 cells (2.0 × 10^3^ cells/well) were plated in 96-well plates in 10% FBS–RPMI-1640 for 24 h (Ty-82) or 72 h (MP57) after siRNA transfection, cultured under hypoxia or normoxia for 6 h (Ty-82) or 24 h (MP57) and then irradiated. After the exposure to different doses of radiation, the plates were incubated under normoxia for 48 h (Ty-82 cells) or 96 h (MP57 cells) before calculation of cell viability (absorbance).

### Statistical analysis

Survival rates were estimated using the Kaplan–Meier method, and differences in survival between subgroups were compared using the log-rank test. The associations between CA9 expression and clinicopathological factors as well as *in vitro* data were analyzed using the Student *t-*test, the Chi-square test, and analysis of variance. *P* values are two-sided, and the level of significance was set to < 0.05. The analyses were performed using SPSS Statistics 20 statistical software (Dr. SPSS II for Windows; standard version 20.0; SPSS Inc., Chicago, IL, USA), and “R” software (version 3.4.1; R Development Core Team 2017, A Language and Environment for Statistical Computing, R Foundation for Statistical Computing, Vienna, Austria; http://www.r-project.org).

## SUPPLEMENTARY MATERIALS FIGURES


