# Supplementary material for: Carbonic anhydrase 9 expression is associated with poor prognosis, tumor proliferation, and radiosensitivity of thymic carcinomas
Source: Oncotarget. 2019 Feb 12;10(13):1306–19. doi: 10.18632/oncotarget.26657 (PMC6407679; doi:10.18632/oncotarget.26657)
Supplement: Supplementary file 1 [file oncotarget-10-1306-s001.pdf]

Carbonic anhydrase 9 expression is associated with poor prognosis, tumor proliferation, and radiosensitivity of thymic carcinomas

SUPPLEMENTARY MATERIALS

A

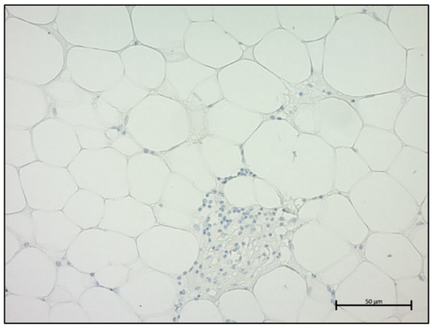

B

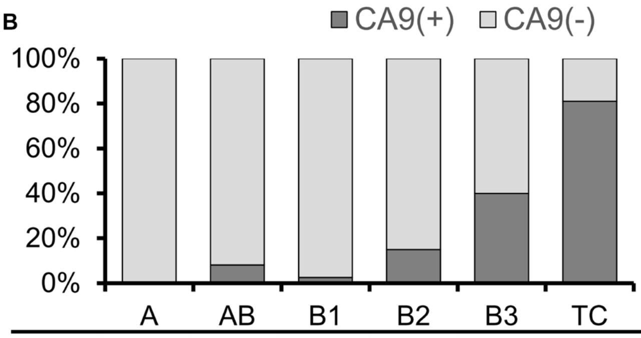

	A (N=17)	AB (N=37)	B1 (N=39)	B2 (N=40)	B3 (N=25)	TC (N=21)	total (N=179)
CA9(-)	17 (100%)	34 (91.9%)	38 (97.4%)	34 (85%)	15 (60%)	4 (19.0%)	142 (79.3%)
CA9(+)	0 (0%)	3 (8.1%)	1 (2.6%)	6 (15%)	10 (40%)	17 (81.0%)	37 (20.7%)

C

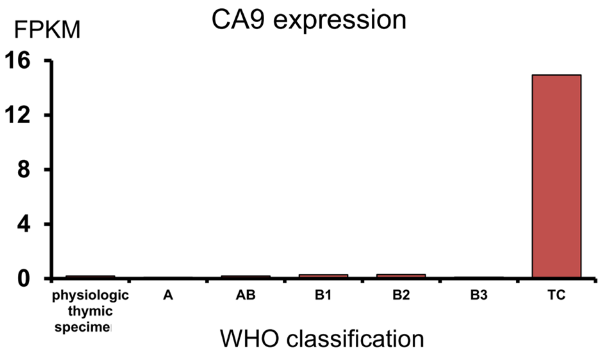

Supplementary Figure 1: (**A**) Immunohistochemical analysis of CA9 expression in physiologic thymic specimens. CA9 was not detected. (**B**), CA9 expression and histological subtypes of TETs. The rates of tumors positive for CA9 expression increased with an increase of the histology determined malignancy grades of TETs. (**C**), The average values of *CA9* mRNA expression according to histological subtypes in TETs. *CA9* mRNA expression determined using NGS significantly correlated with histology. FPKM, Fragments Per Kilobase of exon per Million mapped fragments.

A

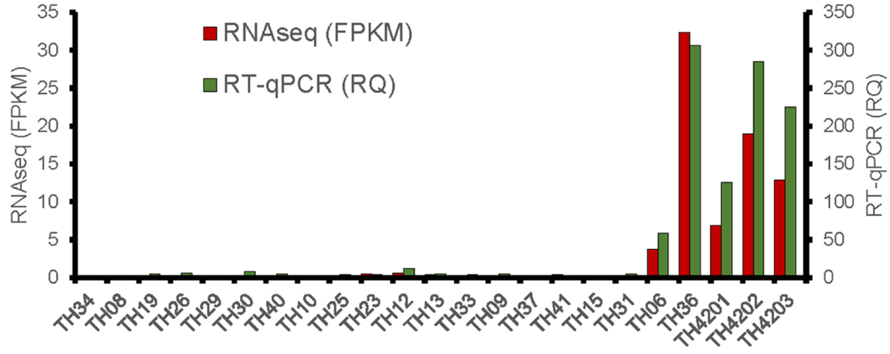

B

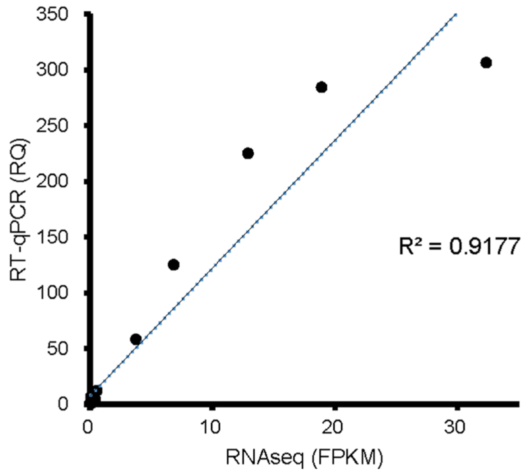

Supplementary Figure 2: Correlation between RNAseq data and RT-qPCR data. (**A**), High *CA9* expression was observed only in TC samples both in RNAseq and RT-qPCR. RQ value was standardized to TH34. (RQ value of TH34 was set as 1) (**B**), The expression values strongly correlated with each other.
